# High temperature injury and auxin biosynthesis in microsporogenesis

**DOI:** 10.3389/fpls.2013.00047

**Published:** 2013-03-11

**Authors:** Atsushi Higashitani

**Affiliations:** Graduate School of Life Sciences, Tohoku UniversitySendai, Japan

**Keywords:** anther development, *Arabidopsis*, auxin, barley, high temperature injury, male sterility, tapetal degeneration, YUCCA

## Abstract

Plant reproductive development is more sensitive than vegetative growth to many environmental stresses. High temperature (HT) injury is becoming an increasingly serious problem due to recent global warming. In wheat, barley, and other crops, the early phase of anther development is most susceptible to HT. I and my colleagues recently demonstrated that HT causes cell proliferation arrest and represses auxin signaling in a tissue-specific manner in the anther cells of barley and *Arabidopsis*. HT also caused comprehensive alterations in transcription. The application of auxin at the same time blocked the transcriptional alterations, led to the production of normal pollen grains, and restored the normal seed setting rate under increasing temperatures. Although synthetic auxins have been used widely as potent and selective herbicides, these recent results indicate that auxin is useful for the promotion of fertility and maintenance of crop yields under the threat of global warming.

## GLOBAL WARMING THREATENS PLANT REPRODUCTION

Plants are highly adaptable and display phenotypic plasticity in response to environmental changes. These characteristics are typically observed during vegetative growth. By contrast, early reproductive growth is more vulnerable to environmental stress (Wollenweber et al., 2003; Läuchli and Grattan, 2007). Therefore, the climate change associated with recent global warming threatens plant reproduction (IPCC, 2007). Lobell and Field (2007) reported that, for wheat, maize, and barley, there was clearly a negative correlation between worldwide crop yields and recent warming. Moderately elevated temperature (up to 5–10°C higher than normal temperature) principally influences the early phase of anther development by causing a reduction in pollen. This leads to a failure in fertilization and a reduction of seed set, which have been observed in many plant species including wheat, barley, tomato, cowpea, and *Arabidopsis* (Saini et al., 1984; Ahmed et al., 1992; Peet et al., 1998; Sakata et al., 2010b; Kim et al., 2001; Sakata and Higashitani, 2008). In this mini review, I introduce the current knowledge of the molecular and physiological mechanism(s) underlying moderately high temperature (HT) injury.

## EFFECT OF INCREASING TEMPERATURES ON ANTHER EARLY DEVELOPMENT

In wheat, it has been reported that two types of abnormal microsporogenesis occur in response to HT stress at the onset of meiosis (Saini et al., 1984). The first type is the occurrence of premature tapetal degeneration during meiosis and the abortion of pollen grain mitosis 1 (PGM1). The resultant plants exhibit a complete loss of spikelet fertility (Saini et al., 1984). The second type is the completion of PGM1 in all microspores but the failure of many microspores to complete PGM2. The anthers of the resultant plants contain a mix of fertile and sterile grains (Saini et al., 1984). The double-rowed barley plant (*Hordeum vulgare* L. cv. “Haruna-nijyo”) is an effective model plant for studies of floral development, and reveals both the first and second types of abortions observed in wheat under HT conditions (Sakata et al., 2010b; Abiko et al., 2005; Oshino et al., 2007). The panicle of the main stem grows to a length of approximately 1 mm at the four-leaf stage (when the tip of the fourth leaf has emerged). At the five-leaf stage, the panicle becomes approximately 2–3 mm in length, and each spikelet develops three stamen primordia and one pistil primordium. During the subsequent 5 days beginning at the five-leaf stage, the panicles grow to approximately 10 mm in length, and pollen mother cells (PMCs) and tapetum cells are formed in the anthers (Sakata et al., 2010b; Abiko et al., 2005). During this period beginning at the five-leaf stage, the most severe injury is inflicted by increasing temperatures. Exposure to moderately elevated temperatures (30/25°C day/night) for 5 days disrupts pollen development so that the anthers completely lack pollen grains (Sakata et al., 2010b; Abiko et al., 2005). HT causes abnormal cell proliferation arrest and premature degradation in the developing anther cells (Sakata et al., 2010b; Abiko et al., 2005; Oshino et al., 2007). When the 5 day HT treatment starts at the four-leaf or six-leaf stage, the two types of abortions observed in wheat (described above) appear in barley (Sakata et al., 2010b; Abiko et al., 2005).

## PROLIFERATION ARREST OF ANTHER EARLY DEVELOPING CELLS BY INCREASING TEMPERATURES

In barley, increasing temperatures suppress cell proliferation of anther parietal cells, sporogenous cells, and PMCs in a tissue-specific manner (Abiko et al., 2005; Oshino et al., 2007). DNA replication in mitochondria and chloroplasts stops within 24 h after elevated temperatures, and subsequent nuclear DNA proliferation also is inhibited (Oshino et al., 2011). Transcription of DNA replication-related genes such as DNA replication licensing factor, DNA polymerases, and histone genes are repressed rapidly under HT conditions (Abiko et al., 2005; Oshino et al., 2007, 2011; Sakata et al., 2010a). In developing ovules, seedlings, and culture cells, cell proliferation rates and expression levels of DNA replication-related genes are not altered by moderately elevated temperatures. By contrast, abiotic stress-related genes (heat shock protein genes, pathogen-related genes, superoxide dismutase genes, glutathione *S*-transferase genes) are equally or more strongly upregulated in these organs and tissues compared with the expression levels in developing anthers (Abiko et al., 2005; Oshino et al., 2007). HT generally induces oxidative damage, misfolding of newly synthesized proteins, and denaturation of existing proteins. Therefore, HT accelerates the transcription and translation of heat shock proteins, the production of antioxidants, changes in the organization of cellular structures including organelles, and changes in membrane function (Fitter and Hay, 1987; Weis and Berry, 1988; Ellis, 1990; Gong et al., 1997; Bray et al., 2000; Maestri et al., 2002). These results indicate that the developing anther cells undergo both general and specific transcriptional alterations in response to moderately elevated temperatures, including the silencing of cell proliferation and DNA replication genes.

## PREMATURE PROGRESSION OF ANTHER DEVELOPMENTAL PROGRAM AND FATE UNDER HT CONDITIONS

The development and differentiation of anther cells, including the specification of cell lineage and cell fate, are well-regulated programs. Sporogenous cells differentiate into PMCs and enter meiosis. The four differentiated layers of anther wall cells (epidermal, endothecium, middle layer, and tapetum cells) are sequentially degraded during pollen maturation. This degradation process appears to be controlled by programmed cell death (PCD) and results in dehiscence of anther walls (Varnier et al., 2005). Specific abnormalities occur during this degradation process, such as abnormal mitochondrial morphology, vacuolization, and chromosomal DNA fragmentation (Varnier et al., 2005). A similar type of PCD prematurely occurs during abnormal abortion of anther cells by cytoplasmic male sterility in rice and sunflower plants (Balk and Leaver, 2001; Ku et al., 2003). Exposure to moderately elevated temperatures for 5 days at the five-leaf stage of barley leads to increased vacuolization, swelling of mitochondria, irregularities in the rough endoplasmic reticulum, and the premature degradation of anther wall cells (Oshino et al., 2007, 2011). HT induces increased DNA fragmentation (approximately 180 bp ladders) of chromosomal DNA (Oshino et al., 2007). In addition, nuclear membranes are disrupted and the nuclear density is significantly reduced in PMCs. These results suggest that HT specifically causes premature PCD in anther cells. The timing of expression of several transcripts, including anther-specific lipid transfer protein (LTP) genes, shifts to an earlier stage in response to exposure to HT (Oshino et al., 2007). Crimi et al. (2006) reported the pro-apoptotic effect of maize LTP in an *in vitro* mammalian mitochondrial system. These anther-specific developmental programs and fate might increase the sensitivity of male reproductive development to many environmental stresses.

## INCREASING TEMPERATURES SPECIFICALLY REDUCE ENDOGENOUS AUXINS IN DEVELOPING ANTHERS

The phytohormone auxin orchestrates many physiological and developmental processes including growth control, organ patterning, and root and shoot architecture (Teale et al., 2006). In *Arabidopsis* seedlings, moderately HT stimulates the elongation of hypocotyls by activation of auxin biosynthetic pathways with the tryptophan aminotransferase-encoding gene TAA1/TIR2 (Gray et al., 1998; Yamada et al., 2009). This transcription is positively upregulated by increased temperature in hypocotyls, cotyledons, and root (Yamada et al., 2009). By contrast, endogenous auxin levels specifically decrease in the developing anthers of barley and *Arabidopsis *during the early response to HT conditions (Sakata et al., 2010a). Immunohistochemical analysis using anti-IAA antibodies reveals that endogenous auxin levels are specifically reduced by increasing temperatures in barley early developing anther cells (Sakata et al., 2010a). In an *Arabidopsis *DR5-GUS recombinant line that places the GUS gene under the control of a synthetic auxin response element (Ulmasov et al., 1997), the strongest GUS activity appears in PMCs and tapetum cells at stage 10 of floral development when the microspores are visible in the anthers (Cecchetti et al., 2008; Sakata et al., 2010a). In recombinants exposed to moderate HT (31 or 33°C) for 1 day, the GUS signals significantly decreased in an anther-specific manner (Sakata et al., 2010a,b). The expression of the *YUCCA *auxin biosynthesis genes is repressed by HT in both barley and *Arabidopsis* (Sakata et al., 2010a). *YUCCA *flavin monooxygenases are implicated in the biosynthesis of indole-3-acetaldoxime (IAOx), which is an intermediate for one of several tryptophan-dependent auxin biosynthetic pathways (Zhao et al., 2001). Expression of *YUCCA* genesis temporally and spatially controlled (Cheng et al., 2006; Cecchetti et al., 2008; Hirano et al., 2008). In *Arabidopsis*, double or triple mutants that include *yuc2* and *yuc6 *have a total loss of male fertility and form short stamens that lack pollen grains (Cheng et al., 2006). Given that these mutant phenotypes are quite similar to the phenotypes of HT injury, biosynthesis of endogenous auxin with *YUCCA *in developing anthers is a major factor responsible for the HT-induced abortion of pollen and reduction of auxin. These phenotypes are highly conserved in monocots and dicots. Tissue- and organ-specific auxin biosyntheses independently and differentially control in response to fluctuation of temperatures (Sakata et al., 2010a,b).

## APPLICATION OF AUXIN REVERSES HT INJURY

**FIGURE 1:**
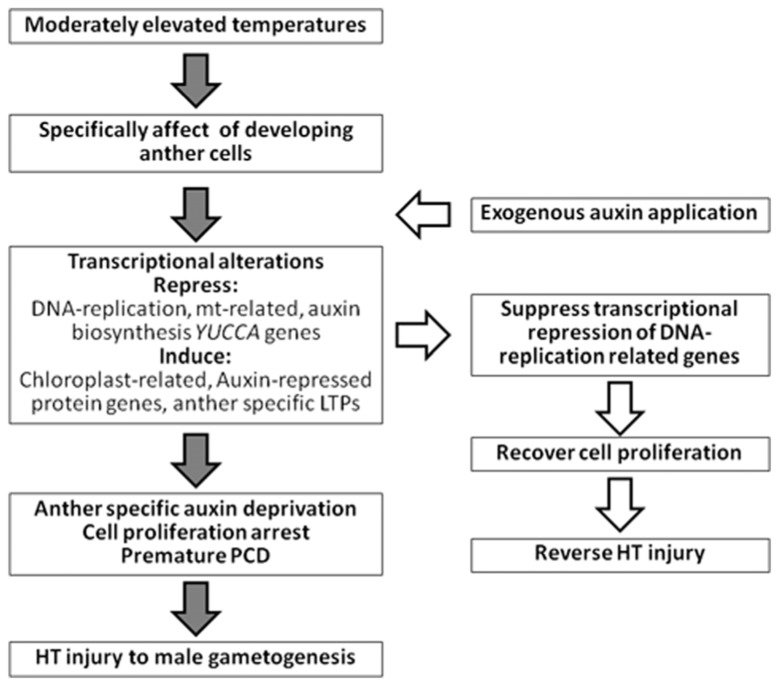
**Summary of HT injury to anther early development and effect of exogenous application of auxin**.

Comprehensive transcriptome analyses using more than 600 microarray datasets of barley show high and positive pairwise correlations between the expression profiles of auxin-induced genes, DNA replication-related genes, and mitochondrial-related genes (Oshino et al., 2011). These genes are negatively correlated with the expression profiles of auxin-repressed protein genes and photosynthesis- or chloroplast-related genes. In developing anthers, HT stress inhibits expression of the former genes and upregulates the expression of the latter genes (Oshino et al., 2007, 2011). Therefore, application of exogenous auxin recovers the expression of DNA replication-related genes under HT conditions (Sakata et al., 2010a; Oshino et al., 2011). Anther cell proliferation and development, and subsequent fertilization and seed setting, are restored under HT conditions by application of exogenous auxin (**Figure 1**). Therefore, HT-tolerant plants might be obtained by controlling anther-specific auxin biosynthesis genes using recombinant DNA technology. The current data indicate that the appropriate application of auxin is useful for the promotion of plant fertility and maintenance of crop yields under the threats of global warming.

## Conflict of Interest Statement

The author declares that the research was conducted in the absence of any commercial or financial relationships that could be construed as a potential conflict of interest.
